# Hematological patients profile admitted to an intensive care unit over 20 years experience

**DOI:** 10.1186/2197-425X-3-S1-A242

**Published:** 2015-10-01

**Authors:** R Fernandez Garda, E Rodriguez Ruiz, R Hernandez Vaquero, E Giraldez Vazquez, A Garrote Freire, JR Fernandez Villanueva

**Affiliations:** Intensive Care Unit, Hospital Clinico Universitario Santiago de Compostela, Santiago de Compostela, Spain

## Objective

To determine Mortality in Hematological patients admitted at the ICU of University Clinical Hospital of Santiago de Compostela from August 1995 to November 2014.

## Material and Methods

Retrospective observational study of long-term Survival of total Hematological patients admitted to the ICU of University Clinical Hospital of Santiago de Compostela between August 1995 and November 2014. 93 patients were enrolled by variables of Age, Gender, APACHE-II Score, Hematological Disease, Claim Inpatient Admission, Length of Stay, Mechanical Ventilation, Continuous Renal Replacement Techniques (CRRT) and Cause of Death.

## Results

93 patients studied, 69% male, with Mean Age 57 years and SD 15 years. Mean APACHE-II were 22 with SD 9.5, 68% had an APACHE-II >16. The most frequent Hematological Disease was Lymphoma (44%) followed by Leukemia (33%), Multiple Myeloma (7.5%), Myelodysplastic Syndrome (5%) and Others (10.5%). The most frequent cause for ICU admission was Acute Respiratory Failure (44%). Mean ICU Stay was14 days with SD 15 days. 76% of patients needed Mechanical Ventilation and 29% were into CRRT. Mortality at ICU was 59%, the most frequent Cause of Death was Multiple Organ Dysfunction Syndrome (29%), followed by evolution of Hematological Disease (15%). In a Subgroup Analysis based on Year of Admission, differences in Mortality rates were noticed before and after 2006 (72% vs 48%) with no statistical significance.

## Conclusions

Mortality at ICU was 59%. Most frequent Cause of Death was Multiple Organ Dysfunction Syndrome (29%) and Hematological Disease (15%). In a Subgroup Analysis based on Year of Admission, differences in Mortality Rates were noticed before and after 2006 (72% vs 48%). Though no statistical significance, we think this notorious difference is based on Hematological treatment improvement and a better Intensive Care support.

